# Next-Generation Sequencing of Cell-Free DNA Extracted From Pleural Effusion Supernatant: Applications and Challenges

**DOI:** 10.3389/fmed.2021.662312

**Published:** 2021-06-14

**Authors:** Ami Patel, Erika Hissong, Lucelina Rosado, Robert Burkhardt, Lin Cong, Susan A. Alperstein, Momin T. Siddiqui, Hyeon Jin Park, Wei Song, Priya D. Velu, Hanna Rennert, Jonas J. Heymann

**Affiliations:** ^1^Division of Cytopathology, Department of Pathology and Laboratory Medicine, New York-Presbyterian Hospital-Weill Cornell Medical College, New York, NY, United States; ^2^Division of Molecular and Genomic Pathology, Department of Pathology and Laboratory Medicine, New York-Presbyterian Hospital-Weill Cornell Medical College, New York, NY, United States; ^3^Clinical Genomics Laboratory, Department of Pathology and Laboratory Medicine, New York-Presbyterian Hospital-Weill Cornell Medical College, New York, NY, United States

**Keywords:** next generation sequencing, pleural effusion, effusion supernatant, cell-free DNA, lung adenocarcinoma

## Abstract

Cell-free DNA (cfDNA) extracted from diverse specimen types has emerged as a high quality substrate for molecular tumor profiling. Analytical and pre-analytical challenges in the utilization of cfDNA extracted from pleural effusion supernatant (PES) are herein characterized in patients with metastatic non-small cell lung carcinoma (NSCLC). Pleural effusion specimens containing metastatic NSCLC were collected prospectively. After ThinPrep® (TP) and cell block (CB) preparation, DNA was extracted from residual PES and analyzed by gel electrophoresis for quality and quantity. Libraries were prepared and sequenced with a targeted next-generation sequencing (NGS) platform and panel clinically validated for plasma specimens. Results were compared with DNA extracted from corresponding FFPE samples that were sequenced using institutional targeted NGS assays clinically validated for solid tumor FFPE samples. Tumor (TC) and overall cellularity (OC) were evaluated. Fourteen specimens were collected from 13 patients. Median specimen volume was 180 mL (range, 35–1,400 mL). Median TC and OC on TP slides and CB sections were comparable. Median extracted DNA concentration was 7.4 ng/μL (range, 0.1–58.0 ng/μL), with >5 ng/μL DNA extracted from 10/14 specimens (71%). Mutations were identified in 10/14 specimens, including 1/3 specimens with median molecular coverage <1,000 reads. The minimal detected allelic fraction was 0.6%. NGS was falsely negative for the presence of one driver mutation. No correlation was identified between sample volume or OC, quality or quantity of extracted DNA, or mutation detection. Despite analytical and pre-analytical challenges, PES represents a robust source of DNA for NGS.

## Introduction

Cell-free deoxyribonucleic acid (DNA) has emerged as an alternative to DNA extracted from traditional formalin-fixed, paraffin-embedded (FFPE) tissue for molecular analysis of tumors (1). Cell-free DNA (cfDNA) species are DNA fragments shed into the extracellular environment in either the bloodstream or body fluids as a result of normal cell turnover (1–4). Analysis of tumor-derived cfDNA has primarily evolved with use of cfDNA in the peripheral circulation (ctDNA) (1). Plasma-based testing allows for serial sampling and less artifact from chemical processing and a more accurate representation of tumor heterogeneity and evolution than for traditional FFPE tissue (5–7). More recently, tumor-derived cfDNA from other specimen types, including cerebrospinal fluid, urine, serous effusion fluid, and fine needle aspiration (FNA) supernatant has also been sequenced with favorable results (8–15). Effusion fluid, in particular, is enriched in cfDNA species, making it a perfect medium for molecular testing (12, 16–20). A major advantage of using such specimens is that they are largely derived from patients with unresectable, advanced stage malignancy. Their collection is minimally invasive, and their utilization can spare patients the discomfort and morbidity associated with more invasive procedures. Several studies have demonstrated that effusion specimens represent a robust source of genetic material for tumor profiling (12, 16, 17, 21). However, several important pre-analytical variables in sequencing of effusion-derived cfDNA testing remain unclear. In order to better define these variables, DNA extracted directly from pleural effusion supernatants (PES/ES) from patients with metastatic non-small cell lung carcinoma (NSCLC) have been analyzed using a targeted next-generation sequencing (NGS) platform clinically validated for plasma specimens.

## Materials and Methods

### Specimen Selection

Fresh pleural effusion specimens of volume ≥35 mL and containing metastatic NSCLC were collected prospectively. Stabilization tubes were not used for any sample.

### Specimen Processing

Aliquots of each specimen were used to prepare one liquid-based (ThinPrep®) preparation according to the manufacturer's protocol (Hologic, Inc., Marlborough, MA) and one cell block preparation. Morphologic diagnosis with immunohistochemical analysis, where necessary, was performed as previously described (22). The remaining supernatant was decanted and stored at room temperature (RT) until the case was signed out, at which time it was transported to the molecular pathology laboratory. There, it underwent repeat centrifugation at 2,000 g for 30 min at 4°C, after which it was immediately stored at −80°C until DNA extraction.

### Clinical Data, Pre-analytical Variables, and Morphologic Analyses

The following pre-analytical variables were cataloged for each specimen: volume; appearance; “processing time”; “sampling time”; morphologic appearance; overall cellularity (OC); and tumor cellularity (TC). Specimen volume and appearance were acquired from the laboratory information system (LIS). Specimen “processing time” and “sampling time” were calculated as follows. Processing time was calculated from the date the specimen was received in the cytopathology laboratory to the date the specimen was frozen in the molecular pathology laboratory. Sampling time was calculated from the date the specimen was frozen to the date of DNA extraction. In order to evaluate morphologic appearance, a cytopathologist and a cytotechnologist independently evaluated ThinPrep® (TP) and cell block (CB) slides (when available) for tumor cell cohesion and presence/absence of necrosis. The OC and TC were estimated using the same TP and CB slides. An additional cytopathologist adjudicated any discrepancies. Patient age and sex were also acquired from the LIS, but they were not considered as pre-analytical variables in the final analysis.

### DNA Extraction

DNA was extracted from 4 mL of postcentrifuged supernatant using the MagMAX® Cell Free DNA Isolation Kit (ThermoFisher Scientific, Waltham, MA) according to the manufacturer's guidelines and then eluted in 20–30 μL buffer. Final DNA concentration was quantified using the Qubit® Fluorometer version 3.0 (ThermoFisher, RRID:SCR_018095). Fragment size distribution was analyzed using a 2100 Bioanalyzer (Agilent Technologies, Santa Clara, CA, RRID:SCR_018043).

For FFPE cytologic CB and histologic specimens, DNA was manually extracted as previously described (23). Briefly, after tumor-enriched areas were macrodissected from sections of 5 μm thickness, DNA was extracted using a Maxwell® 16 FFPE DNA kit (Promega Corp., Fitchburg, WI) according to the manufacturer's instructions.

### Library Preparation, Sequencing, and Data Analysis

For DNA extracted from ES, NGS libraries were prepared according to the manufacturer's recommendation for the Oncomine® Lung cfDNA assay (OLcfD, ThermoFisher, RRID:SCR_007834). OLcfD interrogates DNA for single nucleotide variants (SNVs) and insertion-deletion mutations (indels) in 35 amplicons from 11 genes in which identified alterations are of diagnostic, predictive, prognostic, or research-related significance in NSCLC (Supplementary Material). Barcoded libraries were quantified using the Ion TaqMan® Quantitation Kit (ThermoFisher). Although libraries were quantified after a size selection step, the protocol did not include a formal verification of library fragment size. Pooled libraries at 50 pmol concentration were used for clonal amplification on Ion 530™ Kit-Chef chip (ThermoFisher) and sequenced on the Ion S5™ Sequencer (ThermoFisher, RRID:SCR_017984). Raw sequencing data were aligned to Human Genome Build 19 (hg19) and analyzed using Torrent Suite™ version 5.4 with the VariantCaller plugin and Ion Reporter™ version 5.6 with default parameters (ThermoFisher). The Integrative Genomics Viewer (IGV, Broad Institute, Cambridge, MA, RRID:SCR_011793) was used to visually inspect read alignment and variant call quality, and all variants were manually evaluated before reporting.

For DNA extracted from FFPE cytologic CB and histologic specimens, amplicon-based NGS proceeded using either the Ampliseq™ Cancer Hotspot Panel, version 2 (ThermoFisher) or the Oncomine® Comprehensive Panel v2 (ThermoFisher) as previously described (24).

### Statistical Analysis

Statistical analyses were performed with SPSS version 24 (IBM, Armonk, NY, RRID:SCR_019096). Sample distributions were compared using the Wilcoxon rank sum test. Correlation was calculated using Pearson correlation.

## Results

### Specimen Collection

**Figure 1:**
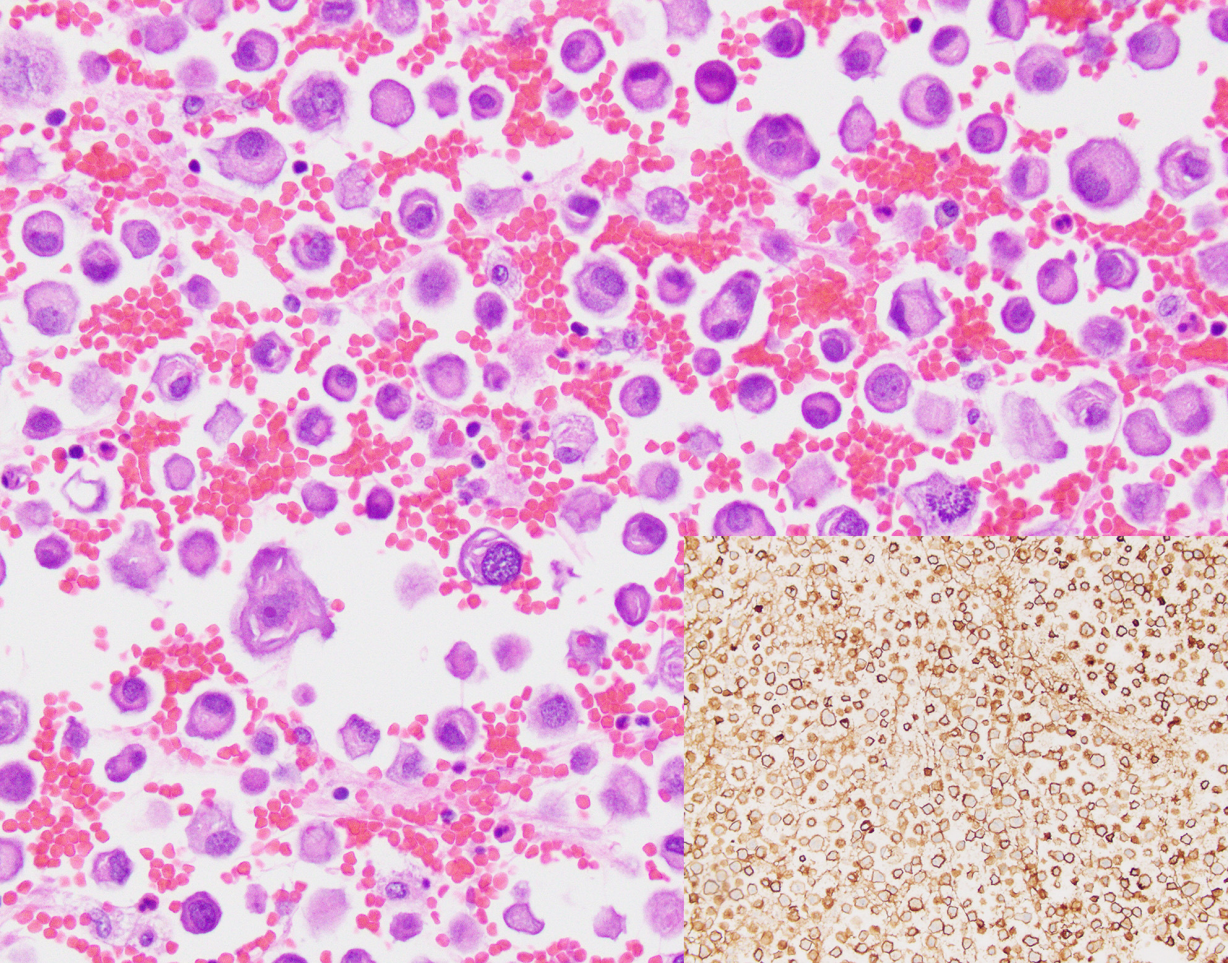
A cell block section of a case (case #10, Table 3) for which overall and tumor cellularity were high (hematoxylin and eosin, 400x). Tumor percentage, quantification of which was aided by immunohistochemical analysis of claudin 4 expression (inset, 400x) was estimated to be 35%.

Forty-one prospectively collected pleural effusion specimens contained metastatic NSCLC. Twenty-seven specimens were excluded due to insufficient volume or collection in CytoLyt®. The remaining 14 fresh pleural effusion specimens were collected from 13 patients (7 women, 6 men) with median age of 72 years (range, 38–92 years, Table 1). Six patients had previously undergone chemotherapy and/or radiotherapy. The median specimen volume was 180 mL (range, 35–1,400 mL). TP and CB slides were available for review for 13 specimens. Median TC on TP slides was 25% (range, <5–>95%). Median TC on the CB slides was 11% with a range of <5–35% (Table 1). Estimation of TC was aided by immunohistochemical analysis of claudin-4 expression, where necessary (Figure 1).

**Table 1:** Clinical data and pre-analytical variables.

**Characteristic**	**Median (Range)**
**Patients (*****n*****=13)**	
Age (years)	72 (38–92)
Sex	
Female	7
Male	6
**Specimens (*****n*** **=** **14)**	
Pre-analytical variables	
Volume (mL)	180 (35–1,400)
Processing time (days)	7 (0–11)
Sampling time (days)	53.5 (5-179)
ThinPrep (*n* = 13)	
Overall cellularity (cells in 10 hpf)	2,000 (0–>20,000)
Tumor cellularity (%)	25 (<5–>95)
Cell block (*n* = 13)	
Overall cellularity (cells in 10 hpf)	841 (150–7,300)
Tumor cellularity (%)	11 (<5–35)

*hpf, high power fields*.

The median processing time was 7 days with a range of 0 to 11 days. The median sampling time was 53.5 days with a range of 5–179 days (Table 1). However, there was an approximately bimodal distribution of sampling times such that 6 specimens had sampling time ≤30 days and 6 specimens had sampling time ≥139 days. There was no correlation between the sampling time and processing time with TC, extracted cfDNA concentration, or fragment size distribution.

### Specimen Cellularity and Morphologic Analysis

**Figure 2:**
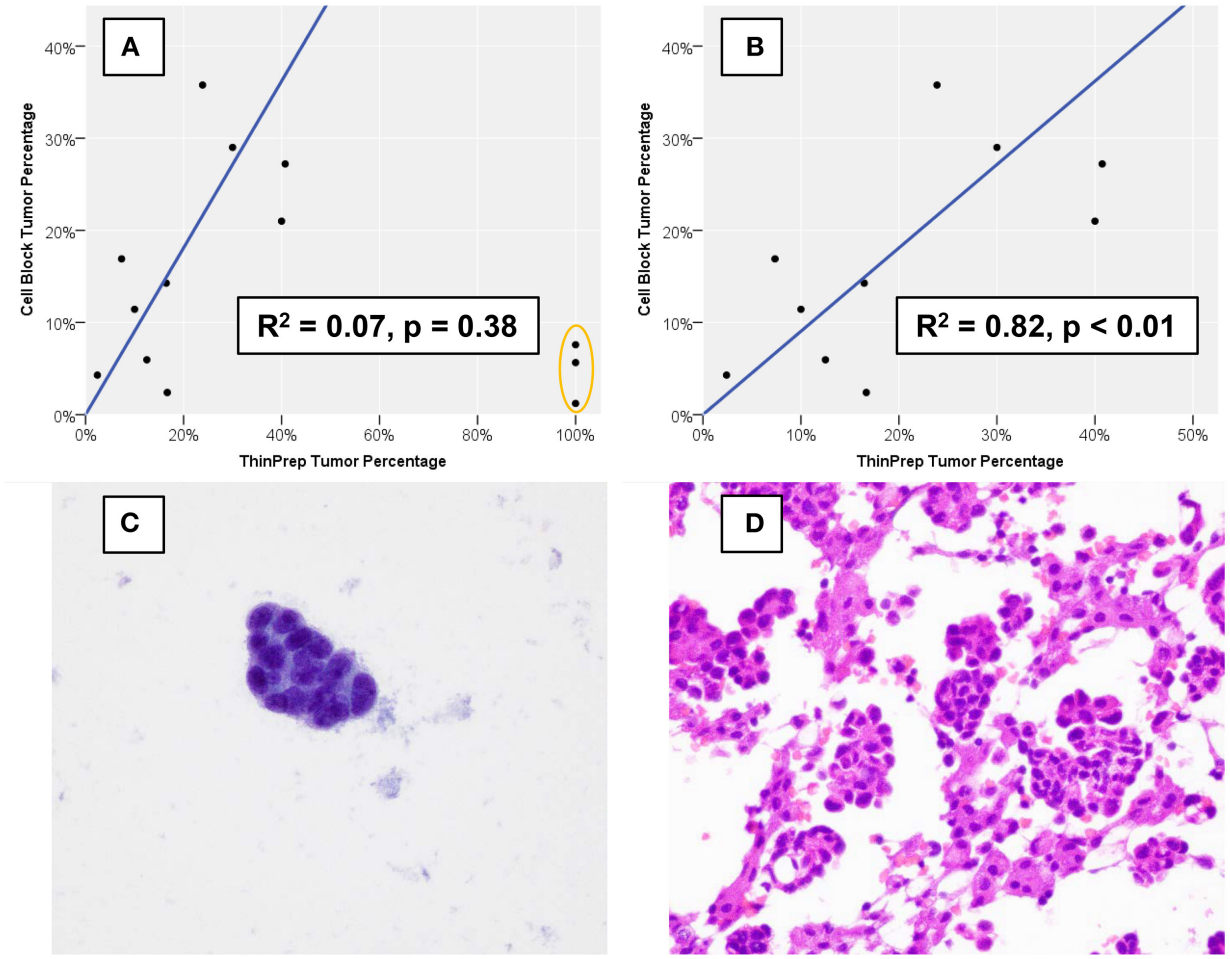
Percentage of nucleated cells that are tumor cells as estimated using formalin-fixed, paraffin-embedded cell block sections vs. using a ThinPrep® slide. **(A)** Three cases (orange oval), all of which demonstrated low overall cellularity on a ThinPrep® slide also demonstrated discordant tumor percentage estimation. **(B)** When the three outlier cases are removed, cell block and ThinPrep® tumor percentages demonstrate a strong and statistically significant linear association. An example of one of the three outlier cases demonstrates an exclusive population of tumor cells **(C)** on the ThinPrep® slide (Papanicolaou stain, 600x), while clusters of tumor cells are intermixed with numerous histiocytes and inflammatory cells **(D)** on cell block sections (hematoxylin and eosin, 600x).

Estimates of TC and OC are provided in Table 1, and estimation of TC demonstrated good agreement when performed for TP slides and corresponding CB sections, with three notable exceptions (Figure 2A). These three specimens, which were all grossly “cloudy” and microscopically markedly hypocellular on the TP slide demonstrated high (>95%) TC on the TP slide but low (<10%) TC on CB sections. For these three specimens, the mutant allelic fraction (MAF) of any identified genetic alteration corresponded better to the cellularity as assessed using CB sections. Upon removal from analysis of these three outlier cases, one of which is represented in Figure 2C (ThinPrep®) and Figure 2D (cell block), median TC on TP slides was 17% (range, <5–40%) and median TC on CB sections was 16% (range, <5–35%). Furthermore, TP TC demonstrated a strong (*R*^2^ = 0.8) and statistically significant (*p* < 0.01) linear association with CB TC (Figure 2B). Importantly, initial cytopathologist and cytotechnologist TC estimates were highly concordant, with adjudication required for only one TP and two CB slides. Upon consideration of all specimens in which a genetic alteration was identified, there was no statistically significant linear association between MAF and TC as assessed on either TP slides or CB sections (data not shown).

Specimens demonstrated a range of morphologic features, including differences in nuclear grade and evidence of gland formation. Notably, tumor cell dyscohesion was slightly more prominent in TP slides than in CB sections, with a predominance of single tumor cells present in six TP slides compared to CB sections (4 cases). Frank morphologic evidence of necrosis was rare with prominent karrhyorectic/karrhyolytic debris present in only 1 case on both the TP slide and CB sections. Interestingly, the quantity of extracted DNA and allelic fraction of the detected mutation in that case were both among the highest in the cohort.

### DNA Extraction

The median extracted DNA concentration was 7.4 ng/μL (range, 0.1–58.0 ng/μL, Table 2), with >5 ng/μL DNA extracted from 10/14 specimens (71%). This quantity of DNA extraction allowed for sequencing input of 20 ng of DNA for all but two specimens. For one of these specimens, sequencing input was 1.3 ng of DNA; for the other, sequencing input was 2.6 ng of DNA.

**Table 2:** Nucleic acid quality control metrics.

**Metric**	**#**
**cfDNA concentration (ng/μL)**	
Median	7.38
Range	0.10–58.00
**Library concentration (pM)**	
Median	445
Range	90–3,118
**Median read coverage**	
Median	42,175
Range	208–127,752
**Median molecular coverage**	
Median	4,619
Range	68–15,798

*cfDNA, cell-free deoxyribonucleic acid*.

**Figure 3:**
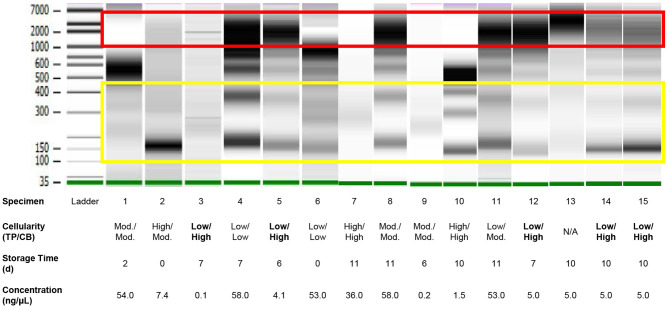
Quality assessment of DNA of variable quantity extracted directly from residual effusion supernatant of variable cellularity and sampling time using gel electrophoresis performed on a 2100 Bioanalyzer (Agilent). High molecular weight DNA (red box) represents cellular/genomic DNA, while low molecular DNA in a ladder pattern in integer multiples of approximately 166 base pairs presumably represents cell-free DNA (yellow box). Intervening bands likely represent a mix of cellular/genomic and cell-free DNA. Cellularities highlighted in bold are those for which estimations—Low, Moderate (“Mod.”), or High—based on review of ThinPrep® slides were discordant from those based on review of cell block sections. Specimen numbers match those in Table 3.

Analysis of DNA fragment size distribution (Figure 3) demonstrated the presence of both high and lower molecular weight DNA in variable quantities. The high molecular weight DNA species presumably represent genomic/cellular DNA, while the lower molecular weight DNA species appear in a ladder pattern of multiples of 166 base pairs (BP), which is characteristic of cfDNA. In the present cohort, relative quantities of DNA species varied widely among samples. In 11 specimens, there was a large proportion of high molecular weight DNA. There was no correlation between the DNA fragment size distribution and history of chemotherapy or radiotherapy, TC (on TP or CB), or detection of a genetic alteration.

### Next-Generation Sequencing

Alterations were detected in 10/14 (71%) specimens, including one specimen (specimen #10, Table 3) out of three with median molecular coverage <1,000 reads. There were also two specimens (specimens #7 and 12, Table 3) in which alterations were identified in both *EGFR* and *TP53*. Overall, the median MAF was 23.1% (range, 0.6–64.7%). The alteration with the lowest MAF—a dinucleotide substitution in *KRAS* (G12F)—was also detected by NGS from FFPE tissue collected in a recent lymph node biopsy.

**Table 3:** Targeted next-generation sequencing results for all specimens.

				**Supernatant NGS results**			**FFPE NGS results**			
**Specimen**	**Age**	**Sex**	**Extracted cfDNA (ng/μL)**	**Gene**	**Variant**	**Allelic fraction (%)**	**Specimen**	**Gene**	**Variant**	**Molecular test**
1	76	F	54.0	N/A	Wild-type		Lung biopsy	*KRAS*	Q61K	ACHPv2
2	60	M	7.4	*KRAS*	G12C	18.5	Pleural biopsy	*KRAS*	G12C	OSH
3	77	F	0.1	N/A	Wild-type		Lymph node	N/A	Wild-type	OCPv2
4	92	F	58.0	*TP53*	R273H	28	Pleural fluid	Indeterminate	None	ACHPv2
5	72	M	4.1	*TP53*	Y205D	5%	Pleural fluid	*TP53*	Y205D	ACHPv2
6^*^	90	M	53.0	N/A	Wild-type		Pleural biopsy	N/A	Wild-type	ACHPv2
7	38	M	36.0	*EGFR*^†^	N771_H773dup	44.3	Pleural biopsy	*EGFR*^‡^	N771_H773dup	Both
8	77	F	58.0	*EGFR*	N771delinsGY	64.3	Lung FNA	*EGFR*	N771delinsGY	ACHPv2
9^*^	90	M	0.2	N/A	Wild-type		Pleural biopsy	N/A	Wild-type	ACHPv2
10	68	M	1.5	*EGFR*	E746_A750del	28.1	Pleural fluid	*EGFR*	E746_A750del	ACHPv2
11	62	F	53.0	*KRAS*	G12F	0.6	Lymph node	*KRAS*	G12F	OCPv2
12	69	F	5.0	*EGFR*^§^	N771_H773dup	8.9	Lung lobectomy	EGFR^**^	N771_H773dup	ACHPv2
13	85	M	5.0	*ERBB2*	Y772_A775dup	64.7	Pleural fluid	*ERBB2*	Y772_A775dup	OCPv2
14	62	F	5.0	*KRAS*	G12D	1.1	Pleural fluid	*KRAS*^††^	G12D	OCPv2
15	62	F	5.0	*KRAS*	G12D	43.4	Peritoneal fluid	*KRAS*	G12D	OCPv2

*cfDNA, cell-free deoxyribonucleic acid; NGS, next-generation sequencing; FFPE, formalin-fixed, paraffin-embedded; N/A, Not applicable; ACHPv2, Ampliseq™ Cancer Hotspot Panel version 2 (DNA only); OCPv2: Oncomine® Comprehensive Panel version 2; OSH, performed at an outside hospital*.

* *Specimens 6 and 9 were collected from the same patient at different time points*.

† *A V337C mutation in TP53 was also detected at an allelic fraction of 31.0%*.

‡ *A concurrent V337C mutation in TP53 was detected*.

§ *An R248Q mutation in TP53 was also detected at an allelic fraction of 4.4%*.

** *A concurrent mutation in TP53 was not detected*.

†† *An additional V197E mutation in TP53 was detected at an allelic fraction of <0.1% upon manual review of sequencing data of concurrent cfDNA*.

The presence of all genetic alterations from 10 cases sequenced from PES were confirmed on sequencing of DNA extracted from FFPE sections of either a concurrent CB (five specimens) or a histologic specimen taken from either the primary (two cases) or metastatic (three cases) site, with two exceptions. In the first specimen (specimen #12, Table 3) both a duplication in exon 20 of the tyrosine kinase domain of *EGFR* (N771_H773dup) with MAF of 8.9% and a deleterious SNV in the DNA-binding domain of *TP53* (R248Q) with MAF of 4.4% were detected in PES. Interestingly, sequencing from FFPE in a histologic lymph node biopsy taken 6 weeks earlier demonstrated the presence of the same *EGFR* alteration but not the *TP53* alteration. The panel (OCPv2) used to sequence the histologic specimen was broad enough to identify alterations in genes not represented in the cfDNA sequencing panel, including a nonsense mutation in *STK11*, as well as amplification of *MYC*, suggestive of genetic complexity. In the second specimen (specimen #4, Table 3), a deleterious SNV in the DNA-binding domain of *TP53* (R273H) at an MAF of 27.7% was detected in PES. Sequencing (ACHPv2) failed using the concurrent pleural fluid FFPE CB. As the patient was ineligible for diagnostic excision and attendant risks of small pulmonary biopsy were deemed to outweigh the benefits, pleural fluid was the only source of DNA on which molecular testing could be successfully performed.

Absence of all genetic alterations in four PES was confirmed using FFPE sections of a FNA CB (one case) or histologic (three cases) specimen taken from the primary site, with one exception (specimen #1, Table 3). It came from a 76 year-old woman who presented with a grossly bloody pleural effusion incidentally identified on routine mammogram. Sequencing (ACHPv2) of DNA extracted from a concurrent histologic lung biopsy specimen showed an activating Q61K mutation in *KRAS* (c.180_181delinsAA). The overall cellularity on both TP and CB was moderate, and, accordingly, ample DNA (54.0 ng/μL) was extracted from PES. However, the TC on both the TP slide and CB sections was low (<5%). The sampling time and processing time (30 and 2 days, respectively) were low compared to other specimens in this study.

There were two specimens for which assay DNA input was <20 ng, and no mutations were identified upon sequencing either specimen. For one of these specimens (specimen #9, Table 3), median molecular coverage was 4,619 reads, and sequencing of PES (specimen #6, Table 3) collected 2 days earlier confirmed the lack of mutations, as did sequencing (ACHPv2) of a concurrent histologic pleural biopsy specimen. For the other specimen (specimen #3, Table 3) associated with low assay DNA input, median molecular coverage was only 178 reads, but absence of mutations was confirmed by sequencing (OCPv2) of a FFPE mediastinal lymph node biopsy specimen procured 1 month earlier.

### Site-Specific Effects

**Figure 4:**
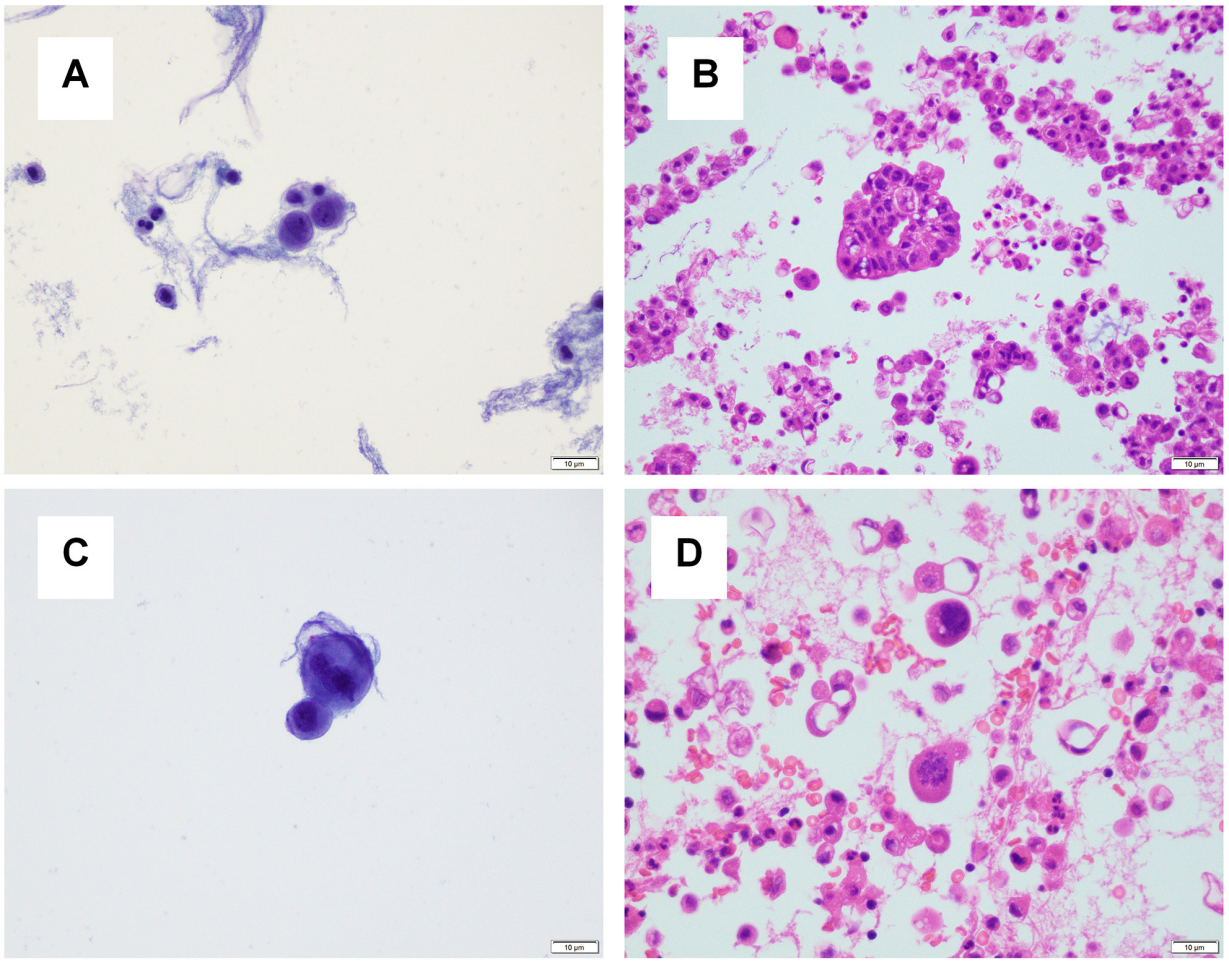
A pleural and peritoneal (ascites) fluid specimen was collected concurrently for one patient. **(A)** The ThinPrep® slide for the pleural fluid specimen demonstrated low overall cellularity, and tumor cellularity was estimated to be 30%. **(B)** A cell block section for the pleural fluid specimen demonstrated high overall cellularity, and tumor cellularity was estimated to be 30%. **(C)** The ThinPrep® slide for the ascites specimen demonstrated low overall cellularity, and tumor cellularity was estimated to be 50%. **(D)** A cell block section for the pleural fluid specimen demonstrated high overall cellularity, and tumor cellularity was estimated to be 5%.

For one specimen (specimen #14, Table 3), sequencing was performed on both residual supernatant fluid and concurrent FFPE CBs of a concurrent peritoneal (ascites) fluid. The fluids were aspirated from the thorax and abdomen of a 62 year-old woman with a history of lung adenocarcinoma metastatic to pleura and peritoneum status post one round of immune checkpoint blockade therapy and multiple failed rounds of chemotherapy who was found to have effusions of increasing size after presenting with shortness of breath. Both the pleural and peritoneal fluid specimens were grossly cloudy and of relatively low volume (90 and 120 mL, respectively). The processing time and sampling time were identical−10 and 5 days, respectively. Morphologic examination of both TP slides demonstrated specimens of low overall cellularity. Conversely, CB sections demonstrated high overall cellularity. Estimated TC for pleural and peritoneal fluid specimens was 30 and 50%, respectively on TP slides; estimated TC for pleural and peritoneal fluid specimens was 30 and 5%, respectively, on CB sections (Figure 4). Accordingly, activating G12D mutations in *KRAS* were identified upon sequencing of pleural and peritoneal ES fluids. However, the MAF of the former was substantially lower than that of the latter (1.1 vs. 43.4%). Sequencing (OCPv2) of concurrent FFPE CBs demonstrated the presence of the same G12D mutations in *KRAS* at MAFs of 9.5 and 40.4%, respectively (Table 4). Additional deleterious SNVs in the DNA-binding domain of *TP53* (V197E) were identified at allelic fractions approximately equal to those of the *KRAS* mutations on sequencing (OCPv2) of concurrent FFPE CBs that were not identified by the automated caller on sequencing (OLcfD) of effusion supernatants (ES). Manual review of the pile-ups in IGV did reveal the presence of the Val197Glu *TP53* mutation in peritoneal and PES at allelic fractions of 0.8 and <0.1%, respectively.

**Table 4:** For two specimens collected from a single patient at a single time point, next-generation sequencing was performed on deoxyribonucleic acid extracted from paired formalin-fixed, paraffin-embedded cell blocks and corresponding effusion fluid supernatants.

**Specimen**	**FFPE cell block**		**Effusion fluid supernatant**	
	***KRAS* G12D (MAF)**	***TP53* V197E (MAF)**	***KRAS* G12D (MAF)**	***TP53* V197E (MAF)**
Pleural effusion	9.5%	9.9%	1.1%	<0.1%
Peritoneal effusion (Ascites)	40.4%	42.1%	43.4%	0.8%

*FFPE, formalin-fixed, paraffin-embedded; MAF, mutant allelic fraction*.

## Discussion

Only a rare study has demonstrated that an NGS platform and methodology clinically validated on plasma specimens can be utilized for malignant PES from patients with metastatic NSCLC (25), with promising results. Our study not only finds PES to be a robust substrate for NGS, it also highlights the challenges involved in the pre-analytic and analytic phases of NGS performed on body fluid cfDNA. Challenges in the pre-analytic phase include optimization of specimen storage and collection, as well as determination of specimen cellularity and nucleic acid quantity and quality. Challenges in the analytic phase include selection of a sequencing methodology with appropriate depth, targets, and interpretation of results as outlined below.

### Specimen Collection

Although guidelines for extracting and processing cfDNA have begun to emerge in recent years (26, 27), investigations have largely focused on ctDNA. Fortunately, guidelines for optimizing plasma specimens for cfDNA testing can inform testing of DNA extracted from other body fluids, including PES, particularly in the pre-analytic phase. For example, cell lysis and release of DNase has been shown to occur during prolonged incubation of blood at RT, even with optimal anticoagulation (1). Reduction of cell lysis would likely reduce the high variation in the relative quantity of DNA species present in our samples. Collection of effusion fluid in preservative or in leukocyte stabilization tubes, neither of which were utilized in the current study, could reduce inter-specimen variability. However, the implications on morphologic and molecular characterization of this method have not been well-characterized in pleural fluid.

The relationship between cfDNA quantity and quality and effusion specimen storage time is not well-established. A previous study of cfDNA extracted from stored and unfixed cytologic material did not report specimen storage times and did not include effusion fluids. For plasma, the American Society of Clinical Oncology (ASCO) and College of American Pathologists (CAP) jointly recommend processing specimens within 6 h of collection (28). Storing plasma supernatant at −80°C for a longer period of time is an acceptable alternative (17), and studies have applied this approach to non-plasma cfDNA samples with good results (17, 19, 25, 29). In the current study, processing time varied greatly between the samples, and no correlation was identified between processing time, sampling time, fragment size distribution or any other variable, including success of NGS. However, the sample size was limited and until a more significant relationship is established between quantity and quality of cfDNA and specimen storage time, it would be advisable to modify laboratory workflows to minimize the latter.

### Specimen Cellularity and Morphologic Analysis

Several factors have been shown to contribute to variation in quantity of cfDNA, particularly that of ctDNA. Environmental exposures, including chemotherapy and radiotherapy can influence levels (1, 30), as can tumor burden, grade, as well as presence/absence of necrosis (1, 31, 32). Although it was associated with an abundance of extracted cfDNA and a high MAF, only one case in the current study demonstrated morphologic evidence of necrosis on both the TP slide and CB sections. To precisely define the range of pre-analytic factors that determine quantity and quality of cfDNA in ES, it would be necessary to examine a more heterogeneous cohort with diverse tumor types, including those associated with a higher rate of cell turnover and necrosis. In this study, overall specimen cellularity did not correlate with quantity of DNA extracted, a non-intuitive finding that, nevertheless, aligns with previous studies.

There is currently no standard method for determining the fraction of cfDNA derived from tumor cells. For FFPE CB and tissue specimens, the estimated fraction of extracted DNA derived from tumor cells is simply derived from quantification of TC. Whether a similar method of estimation is valid for cfDNA is currently unknown (33). Previous studies of cfDNA extracted from ES have estimated TC using liquid-based preparations, CB sections (25), or some combination thereof (13). Upon comparison in a previous study, estimated cellularities were concordant between matched TP slides and CB sections (34). In contrast, for three specimens in the current study, estimated OC and TC were markedly discordant between matched TP slides and CB sections. All these specimens demonstrated both very low OC and very high TC on TP slides. For such specimens, it would be advisable to use CB sections to estimate OC and TC.

### DNA Extraction

The most striking observation in the current study is the wide size variation of DNA fragments extracted from PES. Cell-free DNA fragments in PES have previously been shown to shed in multiples of 166 BP, consistent with nucleosomal structure (17). Many specimens in this study (*n* = 11) and in the literature (13, 17, 35) not only showed characteristic peaks at multiples of 166 BP but also high levels of genomic/cellular DNA. This may indicate low quantities of actual tumor- derived cfDNA—so called “dilutional effect” (36). Indeed, the sole false negative sequencing result (Specimen #1, Table 3) utilized predominantly high molecular weight DNA. Nevertheless, sequencing results utilizing predominantly high molecular weight DNA were confirmed by sequencing FFPE tissue for 10 other specimens. This result is concordant with that from a previous study in which no significant difference in mutational profile or MAF was identified upon hybrid capture-based NGS of fragmented vs. unfragmented DNA extracted from PES of patients with metastatic lung adenocarcinoma (35). Because the current study utilized an amplicon-based sequencing approach without a fragmentation step, the effect of manual DNA fragmentation could not be investigated. There was also no correlation between DNA fragment size distribution and tumor cellularity (on TP or CB), history of chemotherapy or radiotherapy, or detection of a genetic alteration.

### Next-Generation Sequencing

Sequencing platforms vary in the methods of library preparation, signal generation, and variant curation, as well as sequencing targets. Differing results among studies that utilize different sequencing methodologies is to be expected. Studies in which DNA was sequenced by NGS after extraction directly from ES are listed in Table 5. Some previous studies employed sequencing panels with a narrow range of targets (13, 25, 34), including two that, similar to the current study, employed a panel originally designed for ctDNA (12, 25), while other studies employed broad, pan-cancer panels (17, 37). Of note, several studies employed hybrid capture-based sequencing methods (12, 17, 19, 34, 37), which may be more sensitive in the detection of copy number variants (CNVs), and others employed panels that included intronic DNA sequences for the detection of gene fusions (17, 19, 37). The sequencing panel and amplicon-based methodology in the current study did not allow for the detection of either gene fusions or CNVs. As *MET* amplification is an established mechanism and *EGFR* and *ERBB2* amplification are proposed mechanisms of resistance to tyrosine kinase inhibitor (TKI) therapy in *EGFR*-mutated NSCLC (38–40), it would be advisable for laboratories to include an ancillary or secondary mechanism of detection of CNVs for samples of *EGFR*-mutated NSCLC (25). Other mechanisms of resistance to TKI therapy in *EGFR*-mutated NSCLC, including secondary T790M and C797S mutations in *EGFR*, as well as activating mutations in *PIK3CA* may be detected in the current study.

**Table 5:** Review of Studies of next-generation sequencing of nucleic acid extracted directly from residual effusion superanatant fluid.

**References**	**Nucleic acid extraction kit**	**DNA input (ng)**	**Primary site of malignancy**	**Sequencing panel**	**Sequencing panel specificity**	**Minimum allelic fraction detected (%)**
Patel et al. (22)	MagMAX® cfDNA isolation kit^*^	20	Lung	Oncomine® lung cfDNA^*^	Targeted (NSCLC)	0.6
Wei et al. (34)	Gentra puregene® kit^†^	Variable (>20)	Lung	TruSeq™ cancer amplicon^‡^	Not targeted	5
Husain et al. (10)	Plasma/serum circulating DNA maxi kit^§^	80	Multiple	Guardant360® liquid biopsy^**^	Not targeted	Not reported
Roy-Chowdhuri et al. (13)	QIAsymphony® Circulating DNA Kit^†^	10	Multiple	AmpliSeq™ cancer hotspot v2^*^	Not targeted	5.4
Tong et al. (37)	QIAamp® Circulating Nucleic Acid Kit^†^	Variable	Lung	GeneseeqOne pan-cancer^††^	Not targeted	0.1
Guo et al. (19)	QIAamp® Circulating Nucleic Acid Kit^†^	Variable (>50)	Lung	Not reported	Not reported	Not reported
Yang et al. (17)	QIAamp® Circulating Nucleic Acid Kit^†^	120	Multiple	Stanford actionable mutation	Not targeted	1.6
Xiang et al. (25)	MagMAX® cfDNA Isolation Kit^*^	Variable (>20)	Lung	Oncomine® lung cf total nucleic acid^*^	Targeted (NSCLC)	0.3

*cf, cell-free; DNA, deoxyribonucleic acid; NSCLC, non-small cell lung carcinoma*.

* *ThermoFisher Scientific, Waltham, MA*.

† *Qiagen, Hilden, Germany*.

‡ *Illumina, San Diego, CA*.

§ *Norgen Biotek, Thorold, ON*.

** *Guardant Health, Redwood City, CA*.

†† *Geneseeq Technology, Nanjing, China*.

The great advantage of NGS developed for ctDNA, in the setting of sufficient DNA input and great sequencing depth, is the detection of alterations at a very low allelic frequency, which is particularly important in effusion specimens for two reasons. First, some alterations may be subclonal, particularly those that confer resistance to therapy, such as T790M and C797S in *EGFR* that confer resistance to first- and third-generation *EGFR* inhibitors, respectively. As such, they may be present at allelic fractions far below the levels suggested by evaluating cellular tumor fraction. Indeed, Xiang et al. detected several T790M *EGFR* resistance mutations at allelic fractions that were significantly lower (as low as 0.3%) than both the allelic fractions of their corresponding activating *EGFR* mutations and the limit of detection for their FFPE tissue-based assay (25). Second, inflammatory cells and mesothelial cells may vastly outnumber tumor cells in pleural effusion specimens. Indeed, the *median* TC (11%) among our specimens (on CB sections) correlates with that of the *lowest* allelic fraction (5.4%) detected in a previous study (13). Although that study utilized half the DNA input (10 vs. 20 ng in this study), the lowest allelic fraction at which an alteration was detected in the current study (0.6%) is considerably lower. Other studies have shown alterations detected at an allelic frequency of 0.1%; however, quantity of DNA input was not detailed (37). Husain et al. separately utilized a sequencing panel—Guardant360® Liquid Biopsy (Guardant Health, Redwood City, CA)—specifically developed for ctDNA (12). With DNA input of 80 ng, only one SNV was identified in seven samples sequenced, and the authors did not report its allelic fraction. Ultimately, it is advisable that every laboratory in which NGS is performed on ES-derived DNA select and validate a sequencing methodology, panel and quantity of DNA input that allows for detection of alterations present at very low allelic frequency.

The greatest challenge that must be overcome for widespread clinical application of NGS performed on ES-derived DNA is demonstration that it can identify all pathogenic and relevant genetic alterations with great sensitivity. In previous studies, a handful of variants detected by NGS performed on DNA extracted from FFPE were not detected by NGS performed on ES (19, 25, 37). Accordingly, while the paradigm may be shifting (8), nucleic acid extracted from FFPE tissue sections remains the current gold standard substrate for clinical NGS. In the current study, one pathogenic variant was identified upon sequencing of FFPE (the presumptive primary lung mass) but not from a corresponding PES of low TC containing predominantly high molecular weight DNA. Such DNA is presumably of cellular/genomic origin, which may reflect relatively low turnover and release of cfDNA from tumor cells, like that expected from their non-neoplastic mesothelial, histiocytic, and inflammatory effusion fluid counterparts. Given that the assay employed in the current study was able to identify an alteration at an allelic fraction of 0.6%, it is unlikely that the lack of detection was the result of an alteration falling below the limit of detection of the assay. Nevertheless, it would be advisable for pathologists to proceed with caution when performing NGS on predominantly high molecular weight DNA extracted directly from supernatant fluid containing low TC.

Another challenge that must be overcome to allow for widespread clinical application of NGS performed on ES-derived DNA is that of age-related clonal hematopoiesis (ARCH). ARCH is defined as a gradual expansion of hematopoietic stem cell progenitors harboring recurrent, clonal genetic variants in asymptomatic individuals without a diagnosis of hematologic malignancy. The list of variants considered “recurrent” is continuously evolving, as is the threshold for calling such variants (41). Regardless, it is certain that the incidence of ARCH rises with age, and a significant proportion of cfDNA in the peripheral circulation is derived from hematopoietic cells (42, 43). Accordingly, using ultrasensitive hybrid capture-based NGS, Phallen et al. detected potentially ARCH-associated variants with MAF ranging from 0.06 to 7.60% in ctDNA from 37% of a cohort of solid-tumor patients (44). Variants in a single gene (*DNMT3A*) were detected in 93% of those patients' samples, and none harbored variants in any genes included in the panel employed in the current study. However, variants in 4 of the panel genes in the current study—*NRAS, KRAS, BRAF*, and *TP53*—have been estimated to account for up to 5% of ARCH-associated variants (45, 46). Accordingly, Hu et al. demonstrated the presence of ARCH-associated *TP53* variants at MAF ranging from 0.3 to 1.9% upon NGS of DNA extracted from the plasma of 5/33 NSCLC patients (47). Therefore, it is possible that variants of low MAF detected in PES-derived cfDNA may be, in rare cases, derived from contaminating peripheral blood and represent ARCH rather than release of DNA from metastatic epithelial cells, particularly in hemorrhagic effusions from older patients. In the current study, the one case for which a *TP53* alteration was detected in PES but not in DNA extracted from a corresponding (but not concurrent) FFPE specimen was grossly “bloody” specimen and from a patient of moderately advanced age. Its presence could be ascribed to either clonal tumor evolution and/or heterogeneity. However, in the absence of advanced methods of evaluating methylation status or nucleosomal structure or addition of *DNMT3A* to the panel (43, 48), the provenance of the *TP53* alteration cannot definitively be ascribed to metastatic tumor-derived DNA. For the two cases in the current study in which *KRAS* alterations were detected in PES at low MAF, neither of which was grossly “bloody,” the same alteration was identified in DNA extracted from corresponding FFPE specimens.

In addition to challenges, the current study indicates that applications for sequencing of DNA extracted from ES may extend beyond that of current practices. Specifically, previous studies have identified genetic alterations in DNA extracted from ES that were not present in DNA extracted from corresponding FFPE CB sections or tissue biopsies (17, 25, 37). Although no resistance mutations were detected in the current study, a *TP53* alteration was identified upon sequencing of DNA extracted from PES. Library preparation failed using DNA extracted from the concurrent CB sections. This result suggests that, in some cases, DNA extracted from ES may serve to supplement or even supplant DNA extracted from FFPE CB sections.

### Site-Specific Effects

The results of the currently study suggest that the rate and mechanism by which tumor cells shed DNA into effusion fluid requires further study. The results of sequencing of DNA extracted from both supernatant and FFPE CB sections of paired pleural and peritoneal effusion specimens demonstrates differences in allelic fraction of detected alterations. Specifically, while *KRAS* and *TP53* alterations were detected at approximately equal allelic fractions in DNA extracted from FFPE CB sections in both pleural and peritoneal fluid specimens, allelic fractions of *KRAS* alterations in DNA extracted directly from effusion fluid were significantly higher than those of concurrent *TP53* alterations. It is possible that primer competition produced preferential amplification of the *KRAS* amplicon over that for *TP53*, a phenomenon that has been previously described among amplicon-based NGS assays, although to a lesser extent than in the current cases (49). However, the assay utilized to sequence DNA extracted from FFPE was also amplicon-based. Therefore, this result raises the possibility that some DNA species are shed more readily than others by tumor cells, a phenomenon that, to our knowledge has not been previously described.

Furthermore, while allelic fractions of *KRAS* alterations were approximately equal in paired peritoneal effusion (ascites) specimens, the allelic fraction of the *KRAS* alteration detected in DNA extracted from FFPE CB sections from the pleural effusion specimen was far greater than that detected in PES DNA, suggesting differential DNA shedding at different sites. Sequencing of DNA extracted from a greater number of paired ES and FFPE CB specimens will be required to determine the frequency and extent to which DNA species are unequally shed by tumor cells.

In conclusion, DNA extracted from PES represents a robust substrate for NGS developed for plasma specimens, despite pre-analytical and analytical challenges. Reduction of specimen heterogeneity may allow for widespread clinical application of this highly sensitive methodology. Assessment of clonal heterogeneity and site-specific effects on the detection of genetic alterations need further study.

## Data Availability Statement

The datasets generated for this article are not made publicly available due to local data use restrictions. Requests to access the datasets should be directed to Jonas J. Heymann (jjh7002@med.cornell.edu).

## Author Contributions

AP and JH: conceptualization. AP, LC, HP, WS, PV, HR, and JH: methodology and validation. AP, EH, LC, HR, and JH: formal analysis and visualization. AP, LR, LC, HP, HR, and JH: investigation. AP, EH, LR, RB, LC, HP, and JH: data curation. AP, EH, HP, PV, and JH: writing—original draft. SA, MS, WS, HR, and JH: supervision. HP, WS, PV, and HR: resources. LR, MS, WS, HR, and JH: project administration. All authors contributed, reviewed, edited, and approved the submitted version.

## Conflict of Interest

The authors declare that the research was conducted in the absence of any commercial or financial relationships that could be construed as a potential conflict of interest.

## References

[1] Lampignano R Neumann MHD Weber S Kloten V Herdean A Voss T. Multicenter evaluation of circulating cell-free DNA extraction and downstream analyses for the development of standardized (pre)analytical work flows. Clin Chem. (2020) 66:149–60. 10.1373/clinchem.2019.306837/0009-9147 31628139

[2] Jahr S Hentze H Englisch S Hardt D Fackelmayer FO Hesch RD. DNA fragments in the blood plasma of cancer patients: quantitations and evidence for their origin from apoptotic and necrotic cells. Cancer Res. (2001) 61:1659–65. Available online at: https://cancerres.aacrjournals.org/content/61/4/1659 11245480

[3] Stroun M Lyautey J Lederrey C Olson-Sand A Anker P. About the possible origin and mechanism of circulating DNA apoptosis and active DNA release. Clin Chim Acta. (2001) 313:139–42. 10.1016/s0009-8981(01)00665-9/0009-8981 11694251

[4] Leon SA Shapiro B Sklaroff DM Yaros MJ. Free DNA in the serum of cancer patients and the effect of therapy. Cancer Res. (1977) 37:646–50. 837366

[5] Miller AM Shah RH Pentsova EI Pourmaleki M Briggs S Distefano N. Tracking tumour evolution in glioma through liquid biopsies of cerebrospinal fluid. Nature. (2019) 565:654–8. 10.1038/s41586-019-0882-3/0028-0836 30675060 PMC6457907

[6] Coombes RC Page K Salari R Hastings RK Armstrong A Ahmed S. Personalized detection of circulating tumor DNA antedates breast cancer metastatic recurrence. Clin Cancer Res. (2019) 25:4255–63. 10.1158/1078-0432.Ccr-18-3663/1078-0432 30992300

[7] Thierry AR Pastor B Jiang ZQ Katsiampoura AD Parseghian C Loree JM. Circulating DNA demonstrates convergent evolution and common resistance mechanisms during treatment of colorectal cancer. Clin Cancer Res. (2017) 23:4578–91. 10.1158/1078-0432.Ccr-17-0232/1078-0432 28400427 PMC5562356

[8] Roy-Chowdhuri S. Tumor-derived cell-free DNA in body cavity effusion supernatants: a promising alternative for genomic profiling. Cancer Cytopathol. (2020) 128:14–6. 10.1002/cncy.22206/1934-662x 31750996

[9] Siravegna G Marsoni S Siena S Bardelli A. Integrating liquid biopsies into the management of cancer. Nat Rev Clin Oncol. (2017) 14:531–48. 10.1038/nrclinonc.2017.14/1759-4774 28252003

[10] Husain H Melnikova VO Kosco K Woodward B More S Pingle SC. Monitoring daily dynamics of early tumor response to targeted therapy by detecting circulating tumor DNA in urine. Clin Cancer Res. (2017) 23:4716–23. 10.1158/1078-0432.Ccr-17-0454/1078-0432 28420725 PMC5737735

[11] Li YS Jiang BY Yang JJ Zhang XC Zhang Z Ye JY. Unique genetic profiles from cerebrospinal fluid cell-free DNA in leptomeningeal metastases of EGFR-mutant non-small-cell lung cancer: a new medium of liquid biopsy. Ann Oncol. (2018) 29:945–52. 10.1093/annonc/mdy009/0923-7534 29346604

[12] Husain H Nykin D Bui N Quan D Gomez G Woodward B. Cell-free DNA from ascites and pleural effusions: molecular insights into genomic aberrations and disease biology. Mol Cancer Ther. (2017) 16:948–55. 10.1158/1535-7163.Mct-16-0436/1535-7163 28468865

[13] Roy-Chowdhuri S Mehrotra M Bolivar AM Kanagal-Shamanna R Barkoh BA Hannigan B. Salvaging the supernatant: next generation cytopathology for solid tumor mutation profiling. Mod Pathol. (2018) 31:1036–45. 10.1038/s41379-018-0006-x/0893-3952 29463880

[14] Hannigan B Ye W Mehrotra M Lam V Bolivar A Zalles S. Liquid biopsy assay for lung carcinoma using centrifuged supernatants from fine-needle aspiration specimens. Ann Oncol. (2019) 30:963–9. 10.1093/annonc/mdz102/0923-7534 30887015

[15] Ye W Hannigan B Zalles S Mehrotra M Barkoh BA Williams MD. Centrifuged supernatants from FNA provide a liquid biopsy option for clinical next-generation sequencing of thyroid nodules. Cancer Cytopathol. (2019) 127:146–60. 10.1002/cncy.22098/1934-662x 30620446

[16] Yang SR Lin CY Stehr H Long SR Kong CS Berry GJ. Comprehensive genomic profiling of malignant effusions in patients with metastatic lung adenocarcinoma. J Mol Diagn. (2018) 20:184–94. 10.1016/j.jmoldx.2017.10.007/1525-1578 29269277

[17] Yang SR Mooney KL Libiran P Jones CD Joshi R Lau HD. Targeted deep sequencing of cell-free DNA in serous body cavity fluids with malignant, suspicious, and benign cytology. Cancer Cytopathol. (2020) 128:43–56. 10.1002/cncy.22205/1934-662x 31751001

[18] Liu X Lu Y Zhu G Lei Y Zheng L Qin H. The diagnostic accuracy of pleural effusion and plasma samples versus tumour tissue for detection of EGFR mutation in patients with advanced non-small cell lung cancer: comparison of methodologies. J Clin Pathol. (2013) 66:1065–9. 10.1136/jclinpath-2013-201728/0021-9746 23888061 PMC3841772

[19] Guo Z Xie Z Shi H Du W Peng L Han W. Malignant pleural effusion supernatant is an alternative liquid biopsy specimen for comprehensive mutational profiling. Thorac Cancer. (2019) 10:823–31. 10.1111/1759-7714.13006/1759-7706 30779318 PMC6449231

[20] Kawahara A Fukumitsu C Azuma K Taira T Abe H Takase Y. A Combined test using both cell sediment and supernatant cell-free DNA in pleural effusion shows increased sensitivity in detecting activating EGFR mutation in lung cancer patients. Cytopathology. (2018) 29:150–5. 10.1111/cyt.12517/0956-5507 29363841

[21] Leichsenring J Volckmar AL Kirchner M Kazdal D Kriegsmann M Stögbauer F. Targeted deep sequencing of effusion cytology samples is feasible, informs spatiotemporal tumor evolution, and has clinical and diagnostic utility. Genes Chromosomes Cancer. (2018) 57:70–9. 10.1002/gcc.22509/1045-2257 29044880

[22] Patel A Borczuk AC Siddiqui MT. Utility of claudin-4 versus BerEP4 and B72.3 in pleural fluids with metastatic lung adenocarcinoma. J Am Soc Cytopathol. (2020) 9:146–51. 10.1016/j.jasc.2019.12.003/2213-2953 32184064

[23] Baum JE Zhang P Hoda RS Geraghty B Rennert H Narula N. Accuracy of next-generation sequencing for the identification of clinically relevant variants in cytology smears in lung adenocarcinoma. Cancer Cytopathol. (2017) 125:398–406. 10.1002/cncy.21844/1934-662x 28272845

[24] Heymann JJ Yoxtheimer LM Park HJ Fernandez EM Facey KE Alperstein SA. Preanalytic variables in quality and quantity of nucleic acids extracted from FNA specimens of thyroid gland nodules collected in CytoLyt: cellularity and storage time. Cancer Cytopathol. (2020) 128:656–72. 10.1002/cncy.22270/1934-662x 32267620

[25] Xiang C Huo M Ma S Guo L Zhao R Teng H. Molecular profiling for supernatants and matched cell pellets of pleural effusions in non-small-cell lung cancer. J Mol Diagn. (2020) 22:513–22. 10.1016/j.jmoldx.2020.01.011/1525-1578 32036088

[26] Murtaza M Caldas C. Nucleosome mapping in plasma DNA predicts cancer gene expression. Nat Genet. (2016) 48:1105–6. 10.1038/ng.3686/1061-4036 27681289

[27] El Messaoudi S Rolet F Mouliere F Thierry AR. Circulating cell free DNA: preanalytical considerations. Clin Chim Acta. (2013) 424:222–30. 10.1016/j.cca.2013.05.022/0009-8981 23727028

[28] Merker JD Oxnard GR Compton C Diehn M Hurley P Lazar AJ. Circulating tumor DNA analysis in patients with cancer: American society of clinical oncology and college of American pathologists joint review. J Clin Oncol. (2018) 36:1631–41. 10.1200/jco.2017.76.8671/0732-183x 29504847

[29] Hummelink K Muller M Linders TC Van Der Noort V Nederlof PM Baas P. Cell-free DNA in the supernatant of pleural effusion can be used to detect driver and resistance mutations, and can guide tyrosine kinase inhibitor treatment decisions. ERJ Open Res. (2019) 5:16-2019. 10.1183/23120541.00016-2019/2312-0541 30918895 PMC6431750

[30] Meddeb R Pisareva E Thierry AR. Guidelines for the preanalytical conditions for analyzing circulating cell-free DNA. Clin Chem. (2019) 65:623–33. 10.1373/clinchem.2018.298323/0009-9147 30792266

[31] Cristiano S Leal A Phallen J Fiksel J Adleff V Bruhm DC. Genome-wide cell-free DNA fragmentation in patients with cancer. Nature. (2019) 570:385–9. 10.1038/s41586-019-1272-6/0028-0836 31142840 PMC6774252

[32] Diehl F Schmidt K Choti MA Romans K Goodman S Li M. Circulating mutant DNA to assess tumor dynamics. Nat Med. (2008) 14:985–90. 10.1038/nm.1789/1078-8956 18670422 PMC2820391

[33] Pantel K Diaz LA Jr. Polyak K. Tracking tumor resistance using 'liquid biopsies'. Nat Med. (2013) 19:676–7. 10.1038/nm.3233/1078-8956 23744147

[34] Wei S Lieberman D Morrissette JJ Baloch ZW Roth DB Mcgrath C. Using “residual” FNA rinse and body fluid specimens for next-generation sequencing: an institutional experience. Cancer Cytopathol. (2016) 124:324–9. 10.1002/cncy.21666/1934-662x 26682952

[35] Yu Y Qian J Shen L Ji W Lu S. Distinct profile of cell-free DNA in malignant pleural effusion of non-small cell lung cancer and its impact on clinical genetic testing. Int J Med Sci. (2021) 18: 1510–8. 10.7150/ijms.52306/1449-1907 33628109 PMC7893565

[36] Franczak C Filhine-Tresarrieu P Gilson P Merlin JL Au L Harlé A. Technical considerations for circulating tumor DNA detection in oncology. Expert Rev Mol Diagn. (2019) 19:121–35. 10.1080/14737159.2019.1568873/1473-7159 30648442

[37] Tong L Ding N Tong X Li J Zhang Y Wang X. Tumor-derived DNA from pleural effusion supernatant as a promising alternative to tumor tissue in genomic profiling of advanced lung cancer. Theranostics. (2019) 9:5532–41. 10.7150/thno.34070/1838-7640 31534501 PMC6735385

[38] Yu HA Arcila ME Rekhtman N Sima CS Zakowski MF Pao W. Analysis of tumor specimens at the time of acquired resistance to EGFR-TKI therapy in 155 patients with EGFR-mutant lung cancers. Clin Cancer Res. (2013) 19:2240–7. 10.1158/1078-0432.Ccr-12-2246/1078-0432 23470965 PMC3630270

[39] Bean J Brennan C Shih JY Riely G Viale A Wang L. MET amplification occurs with or without T790M mutations in EGFR mutant lung tumors with acquired resistance to gefitinib or erlotinib. Proc Natl Acad Sci USA. (2007) 104:20932–7. 10.1073/pnas.0710370104/0027-8424 18093943 PMC2409244

[40] Camidge DR Davies KD. MET copy number as a secondary driver of epidermal growth factor receptor tyrosine kinase inhibitor resistance in EGFR-mutant non-small-cell lung cancer. J Clin Oncol. (2019) 37:855–7. 10.1200/jco.19.00033/0732-183x 30811294 PMC6455716

[41] Jaiswal S Fontanillas P Flannick J Manning A Grauman PV Mar BG. Age-related clonal hematopoiesis associated with adverse outcomes. N Engl J Med. (2014) 371:2488–98. 10.1056/NEJMoa1408617/0028-4793 25426837 PMC4306669

[42] Acuna-Hidalgo R Sengul H Steehouwer M Van De Vorst M Vermeulen SH Kiemeney L. Ultra-sensitive sequencing identifies high prevalence of clonal hematopoiesis-associated mutations throughout adult life. Am J Hum Genet. (2017) 101:50–64. 10.1016/j.ajhg.2017.05.013/0002-9297 28669404 PMC5501773

[43] Moss J Magenheim J Neiman D Zemmour H Loyfer N Korach A. Comprehensive human cell-type methylation atlas reveals origins of circulating cell-free DNA in health and disease. Nat Commun. (2018) 9:5068. 10.1038/s41467-018-07466-6/2041-1723 30498206 PMC6265251

[44] Phallen J Sausen M Adleff V Leal A Hruban C White J. Direct detection of early-stage cancers using circulating tumor DNA. Sci Transl Med. (2017) 9:eaan2415. 10.1126/scitranslmed.aan2415/1946-6234 28814544 PMC6714979

[45] Xie M Lu C Wang J Mclellan MD Johnson KJ Wendl MC. Age-related mutations associated with clonal hematopoietic expansion and malignancies. Nat Med. (2014) 20:1472–8. 10.1038/nm.3733/1078-8956 25326804 PMC4313872

[46] Watson CJ Papula AL Poon GYP Wong WH Young AL Druley TE. The evolutionary dynamics and fitness landscape of clonal hematopoiesis. Science. (2020) 367:1449–54. 10.1126/science.aay9333/0036-8075 32217721

[47] Hu Y Ulrich BC Supplee J Kuang Y Lizotte PH Feeney NB. False-positive plasma genotyping due to clonal hematopoiesis. Clin Cancer Res. (2018) 24:4437–43. 10.1158/1078-0432.Ccr-18-0143/1078-0432 29567812

[48] Snyder MW Kircher M Hill AJ Daza RM Shendure J. Cell-free DNA comprises an *in vivo* nucleosome footprint that informs its tissues-of-origin. Cell. (2016) 164:57–68. 10.1016/j.cell.2015.11.050/0092-8674 26771485 PMC4715266

[49] Samorodnitsky E Jewell BM Hagopian R Miya J Wing MR Lyon E. Evaluation of hybridization capture versus amplicon-based methods for whole-exome sequencing. Hum Mutat. (2015) 36:903–14. 10.1002/humu.22825/1059-7794 26110913 PMC4832303

